# Piktochart

**DOI:** 10.5195/jmla.2018.517

**Published:** 2018-10-01

**Authors:** Tara Julie Brigham

**Affiliations:** Assistant Professor of Medical Education, Mayo Clinic Libraries, Mayo Clinic, Jacksonville, FL

## INTRODUCTION

How do library staff members communicate with their users, community, or administrators? There are many occurrences and modes in which library staff members attempt to reach others. Examples include posters throughout the library, email or social media messages about new resources or classes, or even the traditional library annual report. It could be argued that a library’s reputation is shaped by how well it communicates with its various audiences. One way to ensure communication success with library patrons is to utilize visuals on any content produced by the library. Consider this information:

- According to a 1997 report by the 3M Corporation, visuals are processed 60,000 times faster than text [1].
- According to eye-tracking studies done by the Nielsen Norman Group, users pay attention to information-carrying images. Furthermore, when the images are relevant, users typically spend more time on the images than reading the text on a web page [2].
- Images help individuals remember information. After hearing a piece of information, people will only remember 10% of it 3 days later. If there is a relevant image with the same information, people will retain about 65% of the information 3 days later [3].

Over the last few years, there has been an increase in the number of tools that can help individuals produce visuals and that generate more appealing content than Microsoft Office Clipart. One of these tools is called Piktochart, a web-based application that allows users to create infographics, presentations, and other image-based content. Piktochart is fairly easy to use without any graphic design experience due to its availability of predesigned, themed templates. Piktochart has a variety of pricing plans. This review will touch on what is available for users using the free version and the paid “PRO” version of Piktochart.

## AUDIENCE

**Figure 1:**
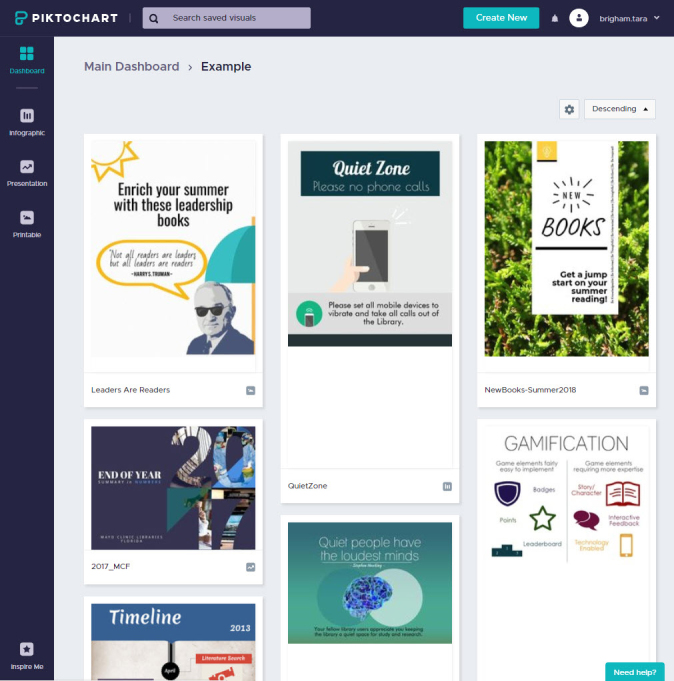
Example of visuals created in Piktochart

This application would be most useful for individuals who are interested in designing visually appealing content for their libraries. Piktochart was helpful when this reviewer needed to create signage that would get the attention of library users but still fall within the design constraints of the organization. Piktochart has also been useful in producing a variety of posters to be used in the library, as well as images and graphs for conference posters and published articles. Additionally, it can assist in creating annual reports for administrators (Figure 1).

## MAJOR FEATURES

One of the most useful features of Piktochart is the predesigned templates. Free users have access to about 40 templates, whereas PRO users can choose from over 600 templates. The templates are organized into 3 different categories: infographic, presentation, and printable. The printable category is further broken down into the areas of posters, reports, and flyers. These templates save time and help busy individuals produce beautiful visuals quickly.

**Figure 2:**
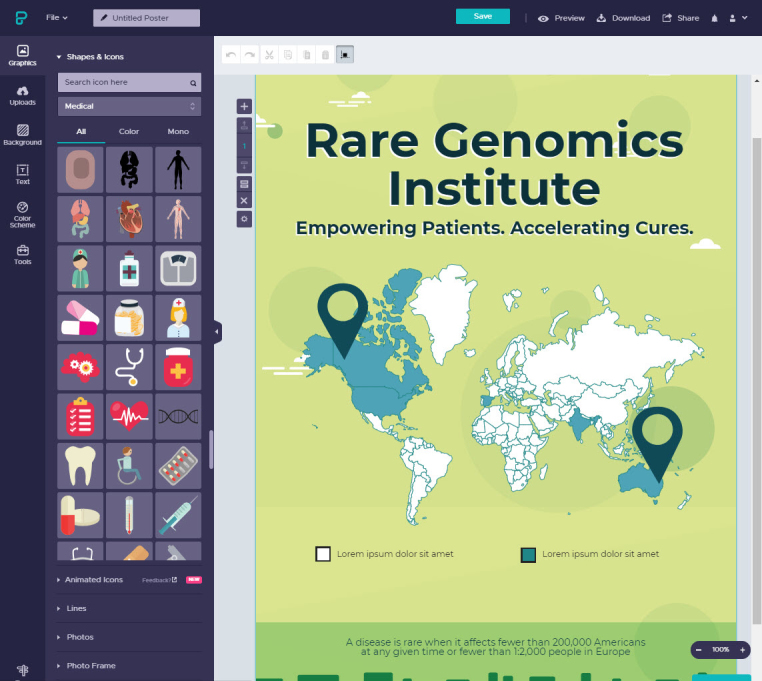
Piktochart editor interface

Piktochart offers a wide variety of icons and graphics for both free and PRO users (Figure 2). These are a major improvement over what individuals have had access to with traditional software programs, such as Microsoft Office Clipart.

Piktochart allows users to upload visuals as well. Free users have 40 megabytes (MB) of storage to upload any JPEG or PNG file; PRO users have 1 gigabyte (GB) of storage and can upload JPEG, PNG, GIF, and SVG files.

Another beneficial feature is the ability for both free and PRO users to insert editable charts, maps, and videos. Charts varying from the typical bar graph to the newer looking “doughnut” can be inserted into any visual. Data for these charts can be entered manually, imported from Google Drive, or imported as a spreadsheet file in the XLS, XLSX, or CLV format. Maps of the world’s regions or countries can also be edited and inserted into any visual. Finally, any link to a YouTube or Vimeo video can be inserted into any infographic, report, or presentation. The inserted video will only work if shared online; the video will not play if the visual was exported and saved as a JPEG, PNG, or PDF file.

## ACCESSIBILITY

To create visuals, Piktochart requires an Internet connection and desktop computer. It does not have a mobile app. It works best with certain browsers, specifically the latest versions of Chrome or Firefox. Because Piktochart uses Javascript, it is not optimized to work with Internet Explorer, Safari, or older versions of Mozilla Firefox. Once the visuals are published online, downloaded, or printed, they can be viewed anywhere.

Piktochart notes that firewalls can block a user from saving or downloading a visual on corporate or shared connections. A network firewall must allow full access to all piktochart.com and create.piktochart.com pages. This may present a problem to staff at libraries who have increased security measures placed on their Internet usage.

## INTEGRATION

Piktochart provides minimal integration with other products or software. Finished infographics, reports, posters, or flyers can be easily shared as a link on social media platforms (Facebook, Twitter, Google+, or Pinterest) or embedded on a website; however, users will more likely want to share the actual visual in a social media posting. This process is a little more cumbersome in that it requires the user to download the visual first and then upload it to the social media platform. Additionally, although Piktochart offers users the option to create slide presentations, they can only be transferred to other applications (e.g., Microsoft PowerPoint, SlideShare, and Evernote) in PNG or PDF file format. It is not yet possible to export Piktochart presentations to Microsoft PowerPoint.

## USABILITY

The Piktochart interface is fairly easy to understand and use. Users will find it helpful to first find a predesigned template and then edit it to fit what they need. Everything works via a drag-and-drop system that makes adding graphics and icons easy. The most frustrating part of working on a visual is that it will never look as good as the predesigned templates; however, additional assistance is available. Piktochart has a “Need help?” button at the bottom of every page, a large Knowledge Database/Support Center with a search box, a YouTube channel that contains short tutorials, and a regularly updated blog with guides and how-tos.

## PRICING

Piktochart has 4 main pricing options: Free, Lite, PRO, and PRO Team. Users can pay on a monthly or annual basis. If paying annually, users can save up to 25%. For those working in the education field and at nonprofit organizations, there is specialized pricing for the PRO and PRO Team versions.

- Free version: Users can create an unlimited number of visuals and have access to approximately 40 templates. Free users can store 40 MB of uploaded images, and the created visuals can be downloaded as a JPEG or PNG file. This most basic version prevents users from removing the Piktochart watermark at the bottom of the visuals.
- Lite version: Users are charged $15 per month or $150 annually. The Lite version is similar to the free version with the exception that Lite users have access to over 600 templates and have 100 MB worth of storage to upload images. Lite users also cannot remove the Piktochart watermark and can only download visuals as a JPEG or PNG file.
- PRO version: For $29 per month or $290 annually, users have access to the more than 600 PRO templates and more than 4,000 PRO icons, 1 GB of storage for image uploads, custom color schemes, and the use of animated icons and SVG files in their visuals. Further, PRO users can remove the Piktochart watermark and export visuals in HD image and PDF format. Subtle features such as folders in which to organize similar visuals and password protection for published visuals are also included.
- PRO Team version: For as low as $13.50 per user per month (pricing for a team between 11 and 25 people), users have access to all of the PRO features as well as more image upload space and the ability to share and annotate projects. Teams can also create customized team templates and assign roles and permissions on the visuals that they are collaborating on.
- Education and Nonprofit: The discounted price of $39.99 per year for the PRO individual subscription or $39.99 per user per year (pricing for a team of 2–25 people) for the PRO Team subscription provides users with all of the capabilities and access listed above for each of the respective PRO versions.

## OVERALL VALUE

Piktochart is an excellent tool to assist users in designing visually appealing content; it can even help those who claim that they are not creative or “are not good at art.” Piktochart’s predesigned templates provide hesitant users with the support that they need to create beautiful, eye-catching infographics, posters, presentations, reports, and social media content. If a user works at or attends a school, college, or university, the education pricing for Piktochart is quite attractive. Even with a free account, users get access to quite a few templates and graphics to keep things interesting. Library staff who are interested in design or simply want to communicate differently with their patrons, community, or administrators are encouraged to try Piktochart.
